# Synchrotron-Generated Microbeam Sensorimotor Cortex Transections Induce Seizure Control without Disruption of Neurological Functions

**DOI:** 10.1371/journal.pone.0053549

**Published:** 2013-01-14

**Authors:** Pantaleo Romanelli, Erminia Fardone, Giuseppe Battaglia, Elke Bräuer-Krisch, Yolanda Prezado, Herwig Requardt, Geraldine Le Duc, Christian Nemoz, David J. Anschel, Jenny Spiga, Alberto Bravin

**Affiliations:** 1 Centro Diagnostico Italiano, Brain Radiosurgery, Cyberknife Center, Milano, Italy; 2 AB Medica, Lainate, Italy; 3 European Synchrotron Radiation Facility, BP220, Grenoble, France; 4 Istituto Di Ricovero e Cura a Carattere Scientifico Neuromed, Località Camerelle, Pozzilli, Italy; 5 Comprehensive Epilepsy Center of Long Island, St. Charles Hospital, Port Jefferson, New York, United States of America; 6 Department of Physics, University of Cagliari and Istituto Nazionale di Fisica Nucleare, Monserrato, Italy; Johns Hopkins Hospital, United States of America

## Abstract

Synchrotron-generated X-ray microplanar beams (microbeams) are characterized by the ability to deliver extremely high doses of radiation to spatially restricted volumes of tissue. Minimal dose spreading outside the beam path provides an exceptional degree of protection from radio-induced damage to the neurons and glia adjacent to the microscopic slices of tissue irradiated. The preservation of cortical architecture following high-dose microbeam irradiation and the ability to induce non-invasively the equivalent of a surgical cut over the cortex is of great interest for the development of novel experimental models in neurobiology and new treatment avenues for a variety of brain disorders. Microbeams (size 100 µm/600 µm, center-to-center distance of 400 µm/1200 µm, peak entrance doses of 360-240 Gy/150-100 Gy) delivered to the sensorimotor cortex of six 2-month-old naïve rats generated histologically evident cortical transections, without modifying motor behavior and weight gain up to 7 months. Microbeam transections of the sensorimotor cortex dramatically reduced convulsive seizure duration in a further group of 12 rats receiving local infusion of kainic acid. No subsequent neurological deficit was associated with the treatment. These data provide a novel tool to study the functions of the cortex and pave the way for the development of new therapeutic strategies for epilepsy and other neurological diseases.

## Introduction

**Figure 1:**
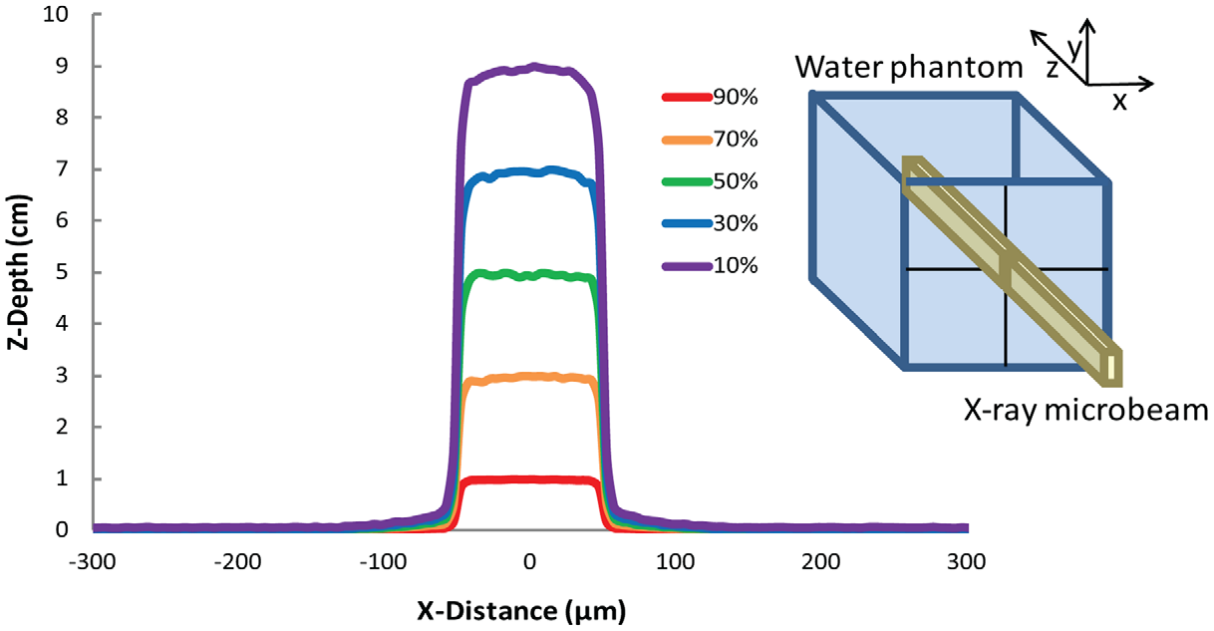
Isodose curves deposited by a microbeam. Dose profiles deposited in a 16×16×16 cm^3^ water phantom by a single 100 µm wide and 10 mm high X-ray microbeam of 100 keV (Monte Carlo simulations), i.e. of energy similar to the median energy of the used spectrum (inset), propagating along the z direction The 50% of the dose is deposited at about 5 cm depth. Objects are not to scale.

Epilepsy is a common neurological disorder with an incidence of 0.3–0.5% in different populations throughout the world. Approximately 20–30% of patients with epilepsy continue to have seizures despite efforts to find an effective combination of antiepileptic drugs. Epilepsy surgery provides an effective tool to ameliorate seizures through the epileptic focus surgical ablation. The resection of large amounts of cortical or hippocampal tissue can be associated to adverse neurological events. The risk to induce severe neurological deficits is particularly high when the epileptic focus involves the eloquent cortex, such as speech or primary motor areas. A non resective surgical technique called multiple subpial transections (MSTs) was developed to treat patients with medically-refractory epilepsy involving eloquent cortex [1]–[2]. This technique requires the placement of vertical incisions through the epileptic cortex in order to cut the horizontal axons responsible of the propagation of seizures while preserving the vertical axons subserving neurological functions [1]–[9]. The vertical columns described by Mountcastle as the basic unit of cortical function [7]–[9] are disconnected but not injured by this approach, allowing the treatment of epileptic foci located over sensorimotor or language cortex not amenable to surgical resection. Several studies have shown the ability of MSTs to stop the propagation of epileptogenic activity and improve seizure control without inducing devastating neurological deficits [1]–[2], [10]–[12]. Synchrotron-generated X-ray beams (microbeams) can induce the equivalent of a surgical cut through the brain tissue by delivering very high doses of radiation (hundreds to thousands of Grays) to tissue slices of microscopic thickness [13]–[14]. In synchrotrons, X-ray beams are tangentially emitted by relativistic electron bunches circulating in a storage ring. The X-ray source is a wiggler (a magnetic structure of alternating poles positioned on a straight section of the storage ring) producing a wide spectrum of photons with an energy range up to several hundreds of kilo-electronvolts (keV). The quasi-laminar and minimally divergent beam can be spatially fractionated into an array of rectangular microbeams of variable size (typically 25–600 µm). Due to an X-ray fluence thousands of times higher than that of standard linear accelerators used in conventional radiotherapy, a dose as high as several hundreds of Grays can be delivered in a fraction of a second. The microbeam ability to reproduce the effects of a surgical cut originates from their peculiar dosimetric characteristics. The X-ray dose delivered to the thin strip of irradiated tissue is reduced by about two orders of magnitude within a submillimetric distance (Fig. 1) [15]–[16]. Therefore, neurons and axons along the penetration path die due to overwhelming doses of radiations (over 100 Gy) while the immediately adjacent tissue is exposed to low doses unable to induce histologically evident tissue damage [17], thus generating vertical incisions separating adjacent cortical columns. This new radiosurgery technique is therefore an attractive experimental tool to induce non-invasive cortical transections.

## Results

**Figure 2:**
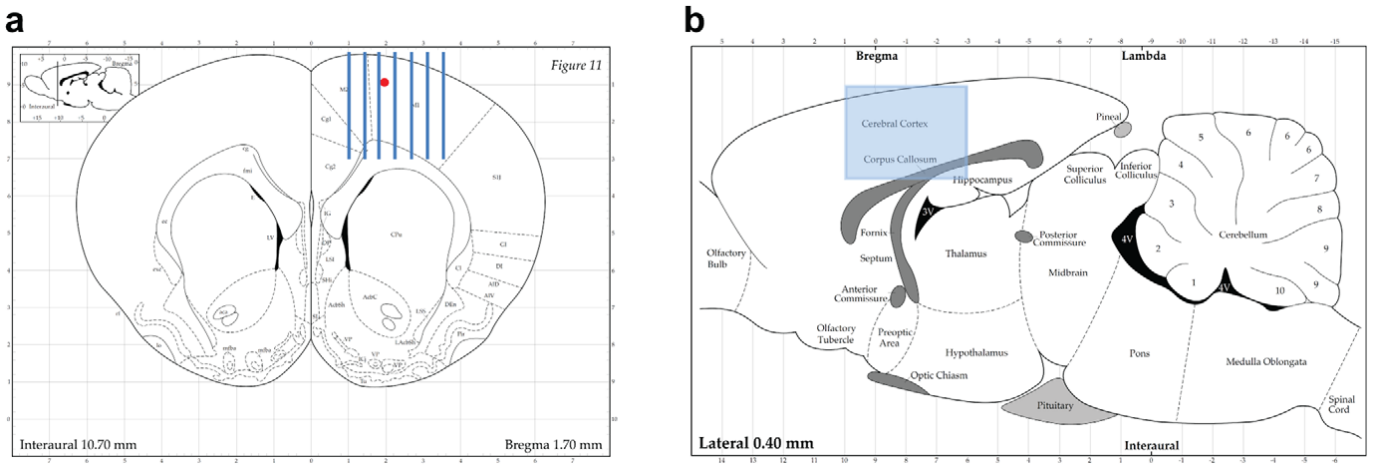
Schematic representation of microbeam irradiation geometry. Coronal (a) and sagittal (b) section of the Paxinos and Watson's rat brain atlas. Arrays of 100 or 600 µm thick microbeams (here represented the 100 µm case, minimum center-to-center spacing: 400 µm) were delivered perpendicular to the sensorimotor cortex using an atlas-based image guided X-ray setup. In (a) is indicated the point where the kainic acid injection was performed to create status epilepticus in rats. Copyright 1998 with permission from Elsevier.

**Figure 3:**
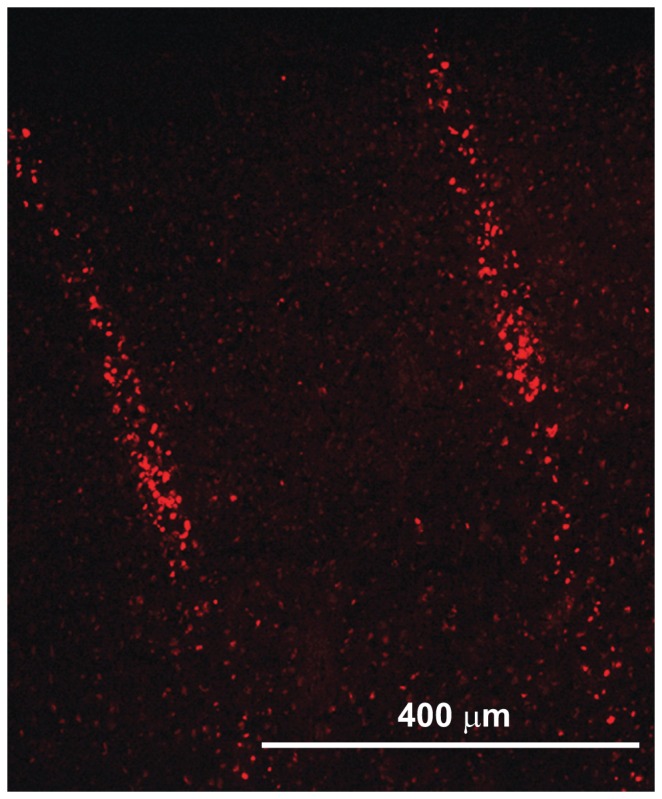
Immunohistochemistry of phosphorylated Gamma-H2AX in the cortex of an irradiated rat. Image shows the microbeam paths across the sensorimotor cortex of healthy rats: apoptotic neurons hit by microbeams (size: 100 µm, c-t-c 400 µm, incident dose 360 Gy) are evident.

**Figure 4:**
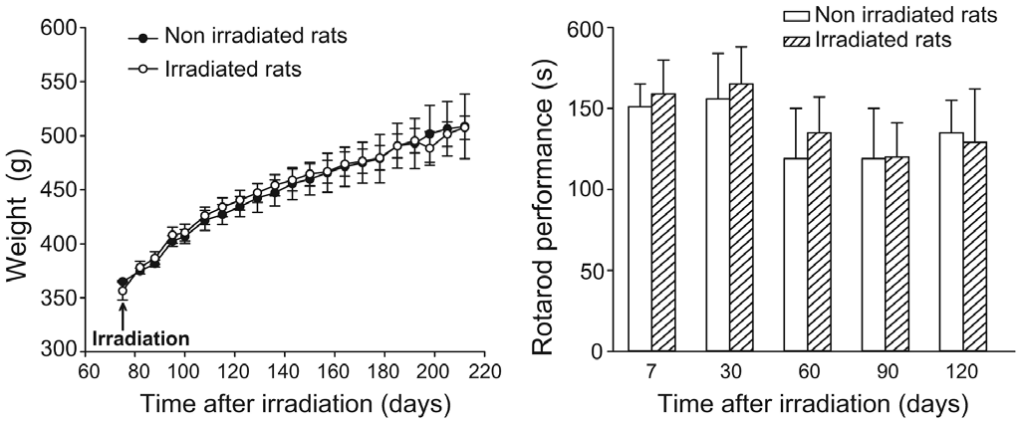
Weight trend and rotarod performance of healthy non-irradiated and irradiated rats. Data are means+S.E.M. (n = 3 for non-irradiated rats and n = 12 for irradiated rats).

**Figure 5:**
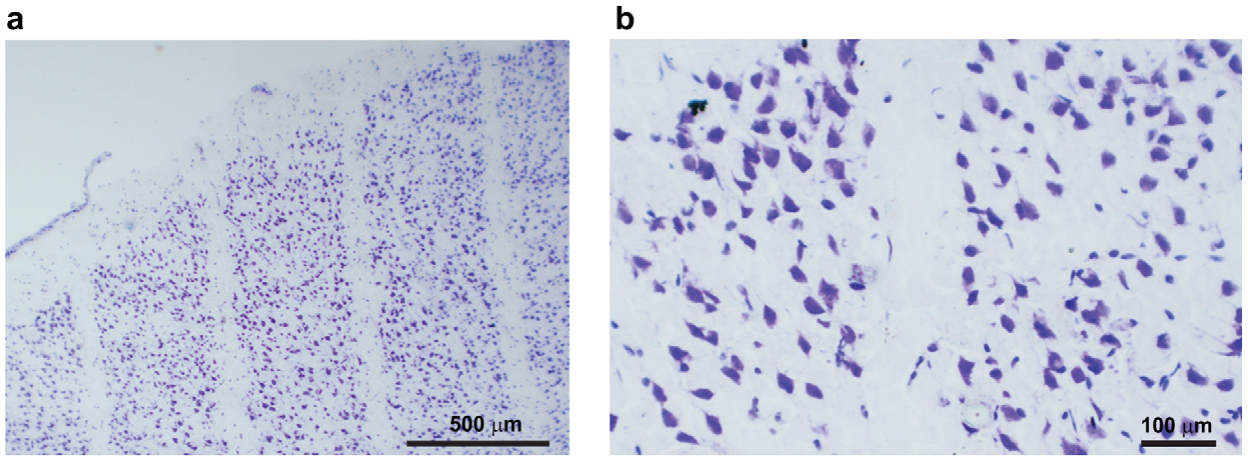
Nissl staining of sensory motor cortex. Nissl analysis performed 7 months after microbeams irradiation shows the lack of neurons along the microbeam path whereas neurons are spared in between microbeams (5b is a higher magnification of 5a).

We evaluated whether microbeam cortical transection could be performed on eloquent cortex without induction of neurological damage in an animal model. We also assessed the efficacy of this approach on seizure control. Microbeam cortical transections were generated in the left motor cortex of 6 healthy two month-old Wistar rats. We explored the effect of two different X-ray microbeam arrays: the sensorimotor cortex of 3 rats was transected by an array of 7 microbeams having a thickness of 100 µm, center-to-center (c-t-c) distance of 400 µm with a skin entrance dose of 360 Gy along the beam trace (peak dose). A second group of 3 rats underwent 4 cortical transections made with 600 µm wide beams spaced by 1.2 mm and depositing a skin entrance dose of 240 Gy (peak dose). The doses delivered to the tissue volume in the middle between the beams (valleys) were limited to 5.3 Gy and 5 Gy, respectively. Beam delivery was done using a rat atlas-based image guided system in anesthetized rats (Fig. 2). Fluorescence immunostaining for phosphorylated H2AX, carried out 24 hours after irradiation, showed a clear-cut microbeam path through the motor cortex (Fig. 3). No acute or chronic neurological or behavioral abnormality was observed after irradiation. Long-term behavioral and neurological monitoring was carried out until sacrifice 7 months after irradiation. These rats showed a regular weight gain and no signs of motor behavior impairment as assessed by the rotarod test (Fig. 4). Histological analysis revealed the absence of neurons along the irradiation path and preservation of the cortical architecture in the immediately adjacent tissue (Fig. 5). To test the hypothesis that cortical column disconnection by microbeam irradiation can provide an effective barrier against the spreading of seizures, we used a rat model of Kainic Acid (KA)-induced convulsive epilepsy. KA is an exogenous agonist of KA ionotropic glutamate receptors which induces severe convulsive seizures following local injection into the sensorimotor cortex [12]. All rats (n = 18) injected with KA developed convulsive seizures (forelimb contralateral or bilateral jerks) within 26±12 min, followed by generalized tonic-clonic seizures and status epilepticus (within 2 hours). Rats were then divided into two groups: control rats (n = 6) and rats receiving microbeam transections (n = 12). Using the same irradiation geometry previously described for healthy rats, transections were induced using either 100 µm wide (n = 6) and 600 µm wide (n = 6) beams. A high dose (HD) and a low dose (LD) protocol were tested for both microbeam groups: peak-valley doses of 360-6 Gy (HD) versus 240-4 Gy (LD) were delivered to rats irradiated with 100 µm wide microbeams, whereas peak-valley doses of 150-6 Gy (HD) and 100-4 Gy (LD) were delivered to rats irradiated with 600 µm wide beams. Dosimetric calculations were performed by using Monte Carlo simulations.

**Figure 6:**
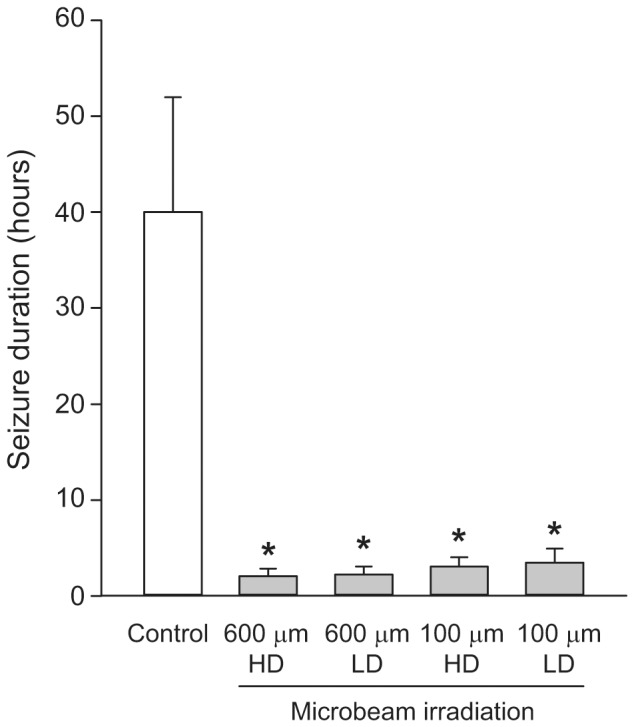
Microbeams irradiations induce a reduction of seizure duration. Duration of convulsive seizures was significantly reduced in rats undergoing transections either with 100 µm wide (n = 6) and 600 µm wide (n = 6) microbeams. A high dose (HD) and a low dose (LD) protocol were tested for both microbeam groups (n = 3 for each of the four irradiated groups and n = 6 for the non-irradiated control group). *p<0.05 (One-way ANOVA+Bonferroni's test) *vs.* non irradiated rats (Control). The differences among the irradiation groups were not statistically significant.

Seizure duration was significantly reduced in rats undergoing transections as compared to control rats (Fig. 6) (p<0.05 -One-way ANOVA+Bonferroni's test- vs. non irradiated control rats). In control non-irradiated rats convulsive seizures disappeared after 40±12 hours. In the group receiving 100 µm transections, convulsive seizures disappeared after an average of 3.0±0.5 hours and 3.3±0.5 hours in the HD and LD protocols, respectively (n = 3 for each group). A similar result was observed in the group of rats treated with 600 µm beams, with seizures disappearing after an average of 2.2±0.2 hours and 2.6±0.5 hours in the HD and LD protocols, respectively (n = 3 for each group). The differences among the irradiation groups were not statistically significant.

## Discussion

Image-guided microbeam radiosurgery is an emerging tool to generate cortical transections or to induce precise focal brain lesions. The Central Nervous System (CNS) radiobiology of microplanar beams was first studied at the National Synchrotron Light Source (NSLS) of the Brookhaven National Laboratory (BNL), where the preservation of CNS architecture after incredibly high radiation doses (up to 4000 Gy) delivered to mouse brain by 25 µm wide beams was first described [18]–[20]. Further work at BNL and the ESRF, investigated the tissue tolerance to microscopic beams at doses tens to hundreds time larger than those allowed by conventional macroscopic beams [21]–[24]. Unidirectional irradiation using microbeam arrays has confirmed through several experiments the exceptional resistance of the normal-tissue to high dose microbeam irradiation [24]–[32], a radiobiological phenomenon referred to as “tissue-sparing effect”, indicating the preservation of the architecture of the tissues traversed by microbeams carrying doses tens to hundreds times higher than those associated with radionecrosis after conventional radiotherapy.

The doses to regenerative normal cells and tissues between microbeams, the “valley” doses, are probably the most important determinants of normal tissue damage in MRT [33]. For a given target, the valley dose depends on three main parameters: the microbeam width, the spacing between the microbeams and the dose delivered along each microbeam. For a given peak dose, the valley dose is higher for wider microbeams and for smaller c-t-c distances. In this pilot experiment microbeam sizes and c-t-c distance were here chosen on the basis of the literature describing the tissue sparing effect of X-ray microbeams deposited in the central nervous system [14], [33]. We have tested combination of microbeam sizes/spacing at the two extremes of the range better documented in the literature, which spans from 25/200 up to 0.68/1200 micron. After having fixed the size and spacing, the peak dose was chosen in order to determine a valley dose between 4 and 6 Gy that is well tolerated by the brain [34]. Immunohistochemical staining using pH2AX show clearly that the neurons hit by the microbeam along its penetration path die almost immediately while the adjacent cells separated by a few micron but outside the high dose volume remain viable (Fig. 3). Progressively lower doses (but still much higher than in conventional radiosurgery or radiation therapy) are required to avoid tissue damage if thicker beams (100 to 600 µm) are used. Submillimetric beams (sized 0.6 to 0.7 mm) appear to retain the tissue sparing effect allowing the delivery of incident doses of 400 Gy to the spinal cord of rats without inducing neurological damage: irradiation of rat spinal cord with four parallel 0.68-mm thick microbeams at 400 Gy in-depth beam dose did not induce paralysis after 7 months in three out of four rats [24]. This latter study showed not only that a highly radiosensitive structure such as the spinal cord can receive a high irradiation dose through a microbeam array without neurologic sequelae but also that a beam width up to 0.68 mm is well tolerated, substantially maintaining the tissue sparing properties of thinner beams. The ability of microbeam arrays to avoid radionecrosis and to preserve the architecture of the irradiated tissue is mainly attributed to the rapid regeneration of normal microvessels. Only a short segment of the microvascular bed receives ablating doses while the adjacent endothelial cells fall into the valley dose region receiving just a few Gy and can restore quickly the continuity of vascular supply [32]–[35]. The wide spatial interface between the unhindered tissue placed in the valleys and the tissue irradiated with peak doses within the microbeam paths facilitates a widespread vascular recolonization of the tissue receiving necrotic doses preventing the dissolution of the architecture of the irradiated tissues [33]. The self-repair of the normal microvasculature through the migration of unaffected cells surrounding the paths of microbeam penetration is considered the most likely basis for this ability of normal tissue to tolerate high dose microbeam irradiation [17], [36]. The tolerance of the vascular bed to high dose microbeam irradiation has been clearly demonstrated by the lack of extravasation of dyes administered to the experimental animals, which remained confined in the vessels after irradiation from 12 h until three months following 1000 Gy [17]. This radioresistance phenomenon was not observed in 9L glioma microvessels, confirming the presence of a differential response in normal and tumour brain tissues in rodents, an effect that can have significant clinical applications [28]. The neoplastic vasculature appears to be unable to replicate the fast repair of the segments hit by the peak dose, facilitating the development of radionecrosis over the irradiated tumor [29], [37].

A novel way to use microbeam arrays in a quasi-surgical way has been explored here: a microbeam array was placed over selected cortical areas in order hit tangentially and cut the horizontal axons connecting adjacent cortical columns. As described above, cortical transections are a surgical procedure to parcellize an epileptic focus located in eloquent cortex [1], [2]. Cutting the horizontal axons required for the spreading of epileptic activity is an effective way to control the seizures without inducing neurologic dysfunction. Synchrotron-generated microbeams can be used to create cortical transections in rats offering a chance to study the tolerance of CNS to this technique. This novel experimental application of microbeams provides a new and attractive tool to modulate cortical function by transecting the fibers connecting the cortical columns. Aside from the tight dosimetry, the low energy of monochromatic beams makes them well suited to treat superficial targets such us the cortex.

The smallest cortical area capable to sustain synchronous spikes generating seizures is estimated to be around 1 cm^2^ in monkeys [38] and 0.5 mm^2^ in rats [39]. It has been shown that parallel cortical transections with a thickness of 25 µm delivering doses up to 1000 Gy are not associated with short or long-term injury to the adjacent tissue [17]. Therefore microbeam transections appear to be well suited to parcellize and disconnect an epileptic focus, even in small animals. We found here that larger beam size and higher doses were associated with faster relief from convulsive seizures. However all rats undergoing cortical transections recovered from convulsive seizures compared to nontransected controls.

These experiments indicate that submillimetric X-ray beams can modulate seizure spreading in eloquent cortex without inducing evident neurological dysfunction. These results suggest further investigations directed to assess the potential of microbeam transections to modulate cortical functions and to treat focal epilepsy. Microbeam transection, either placed over neocortical seizure foci or through the hippocampus, could prove to be an excellent tool to be added to the current radiosurgical techniques used to control seizures. The development of clinical devices delivering submillimetric beams able to generate cortical transections might add a powerful new tool to the clinical treatment of epilepsy and, more in general, to modulate cortical functions in a wide variety of neuropsychiatric disorders.

## Materials and Methods

### Animals

All operative procedures related to animal care strictly conformed to the guidelines of the French Government with licenses 380324 and A3818510002. Male Wistar rats (250–275 g) were purchased from Charles River Laboratories (L'Asbresle, France). Rats were kept under environmentally controlled conditions (ambient temperature = 22°C, humidity = 40%) on a 12-hour light/dark cycle with food and water ad libitum.

### Irradiation source

Irradiations were performed at the ID17 biomedical beamline of the European Synchrotron Radiation Facility (ESRF, Grenoble, France). Details on the technique and of the related instrumentation are reported in [14]. Briefly, synchrotron X-ray radiation originates tangentially from relativistic electron bunches circulating in a storage ring. The source (wiggler) produces a wide spectrum of X-rays with a median energy of 90 keV and range extending, after filtration using Be (0.5 mm), C (1.5 mm), Al (1.5 mm) and Cu (1.0 mm), from 50 to about 350 keV [15]. The quasi-laminar beam obtained so far is collimated into an array of rectangular microbeams of variable size depending on the chosen collimator slits, which are placed about 42 m from the photon source and 1 m upstream from the head of the experimental animals [40]. At ESRF the X-ray dose rate is about 16000 Gy/s.

### Geometry of the beam arrays and irradiation set up of healthy rats

Rats were placed vertically and fixed by ear bars and teeth on a custom-made Plexiglas stereotactic frame and placed on a Kappa-type goniometer (Huber, Germany), by which the rat could be translated and rotated in front to the fixed horizontal X-ray beam. The beam height was defined by a (520±5) µm tungsten slit, placed at 1 m upstream the animal. Microbeams of different widths (100±5 and 600±5 µm) and number (respectively, 7 and 4) were used to irradiate different groups of rats. The c-t-c spacing was (400±5) µm center-to center (c-t-c) for the (100±5) µm beams and (1200±5) µm for the 600 µm beams. These beams are shaped by a hard X-ray tungsten collimator which allows producing arrays of microbeams with a ±2 µm precision in width [40]. Irradiated cortical field was 4 mm on the antero-posterior direction (1 mm anterior to −3 mm posterior to the bregma) and, respectively, 2.5 mm and 4.2 mm on the lateral direction (starting 1 mm lateral to midline in the first case and from the midline itself in the second case [41]). The X-ray dose rate was directly proportional to the instantaneous electron current circulating in the storage ring, which was continuously monitored. The dose was delivered by vertically moving the Kappa goniometer, at a speed that was inversely proportional to the dose to be delivered. Each irradiation lasted less than 1 s. The vertical irradiation field was determined by the opening-closing of fast shutters, located 7 m upstream the rats and synchronized with the movement of the kappa goniometer [40]. All movements were remotely controlled and irradiation values were preset by the operator before the treatment. The animal immobility during exposure was checked on 3 control screens located in the control hutch. The incoming spatially non-fractionated dose was measured using an ionization chamber and the mid-valley doses were calculated with Monte Carlo simulations.

### Monte Carlo simulations and dose calculations

The Monte Carlo code PENELOPE 2006 [42]–[43] was used for calculating the deposited dose. PENELOPE simulates the coupled transport of photons, electrons and positrons in the energy interval from 50 eV to 1 GeV, and in arbitrary material systems. PENELOPE has been widely used in the medical physics field [43]–[45] and in MRT dose assessment [15], [46]–[47]. The main advantage of this code is a careful implementation of accurate low energy electron cross sections, which are of particular importance in this work. The simulation algorithm is based on a scattering model that combines numerical databases with analytical cross section models for the different interaction mechanisms and is applicable to energies (kinetic energies in the case of electrons and positrons) from a few hundred eV to 1 GeV. It uses a mixed simulation scheme in which hard interactions are simulated collision by collision and small angular deflections and energy losses are treated in a grouped manner. Since the working energy range is a few hundreds of keV, the most relevant interactions are photoelectric effect and Compton scattering. The photoelectric cross sections used in PENELOPE are obtained by interpolation in a numerical table that was extracted from the Lawrence Livermore National Laboratory Evaluated Photon Data Library [48]. Regarding Compton scattering, PENELOPE considers bounding effects and Doppler broadening when simulating Compton interactions. In this work the number of primary photon stories was 2×10^8^ in all the calculations.

### Dose deposition simulation geometry

A rat head phantom was constructed with the geometry package in PENELOPE, as described in [15]. It consists of three concentric ellipsoids whose volumes are 13.00 cm^3^ for the brain, 3.13 cm^3^ for the skull, and 7.15 cm^3^ for the skin; these values have been extracted from MRI images acquired on the rats of the same strain and weight. The use of realistic geometries, instead of water phantoms is essential for a correct assessment of the dose deposition. Peak and valley dose calculation was performed following the different irradiation geometries (microbeam number, spacing and width) used in the *in vivo* experiments.

### Induction of seizures by local kainic acid injection and irradiation parameters

**Figure 7:**
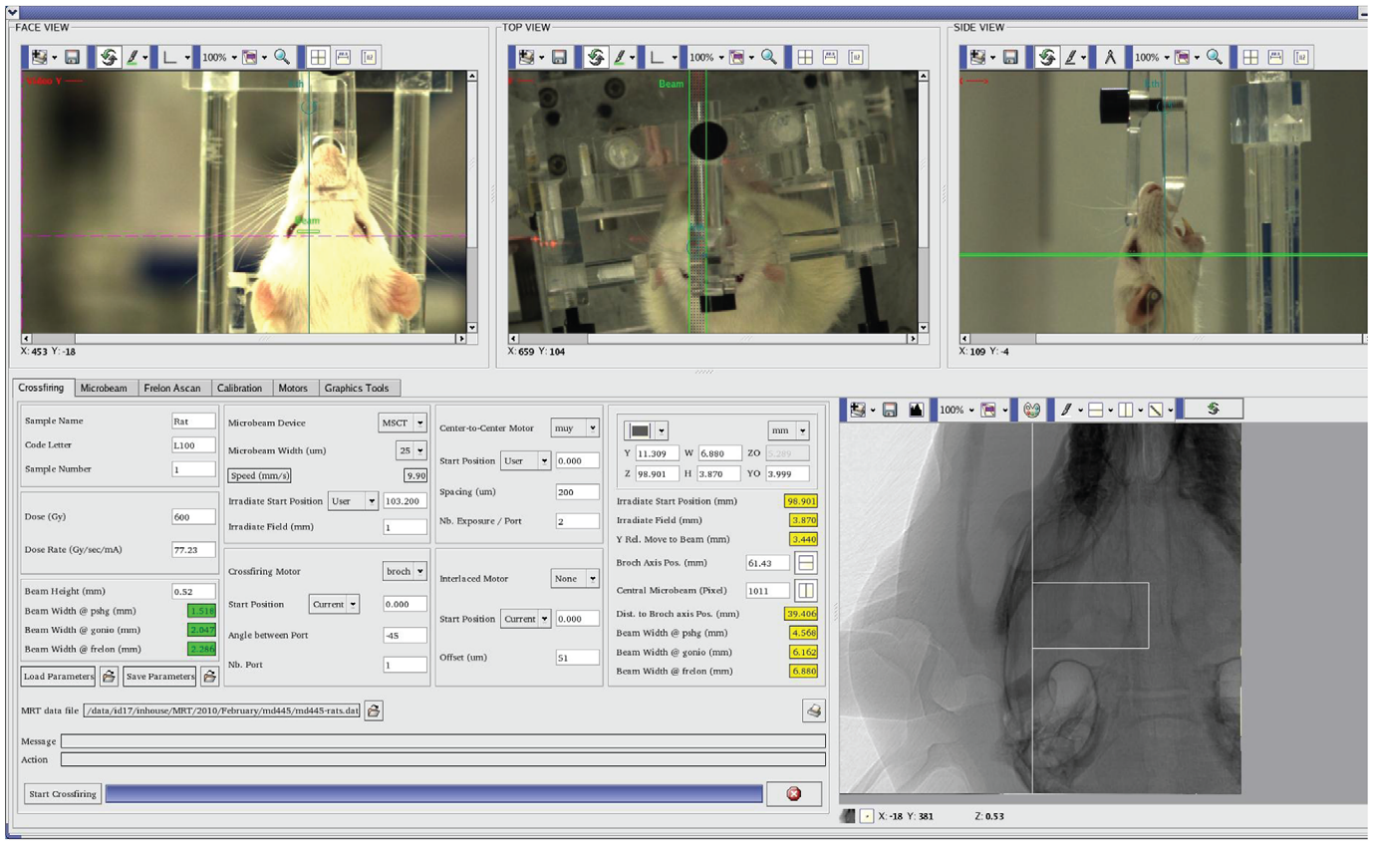
The control panel of the rat irradiation and imaging systems. After an optical prepositioning of the target performed through 3 remote controlled video cameras (three upper panels), high resolution target determination is performed by X-ray imaging. A computer-guided robotic arm (Kappa goniometer, below the rat and not visible in the Figure) moves the stereotactic headframe to acquire radioscopic image necessary to individuate the bregma (radiographic image, bottom right) from which stereotactic coordinates allow the irradiation.

Male Wistar rats were implanted with intracerebral guide cannulas (Bilaney GmbH, Schirmerstr, Germany), under isoflurane (2%) anesthesia, in a Kopf stereotactic frame. The site of implantation was the left cerebral cortex (coordinates: 1.0 mm anterior to the bregma, 2.0 mm lateral to the midline, 1.0 mm ventral from the outer surface of dura mater [41], Fig. 2). KA (20 µg/1 µl) was slowly injected by a microcannula into the left sensorimotor cortex. The cannula was left in place for 2 min before being withdrawn. Rats were allowed to recover in a large heated box for 1 hour and then were transferred to their home cages. Two hours after seizure induction, epileptic activity spread maximally, leading to status epilepticus. At this point the rats were anesthetized and placed on a plexiglas stereotactic frame fixed on the Kappa goniometer to undergo microbeam sensorimotor cortex transections under general anesthesia (ketamine, 100 mg/kg, +xylazine, 10 mg/kg, i.p.). The alignment of the target was performed by in-situ X-ray image guidance [49]. High resolution (pixel size 47 µm) radiographs were performed on rats placed on the goniometer using attenuated X-rays issued from the wiggler source. The bregma was identified on the radiographs allowing positioning the target following the rat atlas [41] (Fig. 7, bottom right). The total dose per image (one image/animal) was <5 mGy and the total imaging time procedure lasted <5 min. The brain cortex was transected using the same irradiation modalities (coordinates, image guidance, microbeam sizes and spacing, doses) described for healthy rats. The transected rats emerged from anesthesia shortly after the procedure. The presence of convulsive seizures allowed to rely on behavioral observation for assessment. Electrocorticography was carried out as well in 3 further rats to verify the feasibility of this technique but, given the possibility of dosimetric distortions induced by the interaction of X-ray beams with the EEG electrodes in the brain, we preferred to rely, during this preliminary experiment, on behavioral observation.

### Evaluation of post-irradiation behavior in normal rats undergoing motor cortex microbeam transections

Twelve rats underwent left motor cortex irradiation. Rats were placed in large transparent Plexiglas boxes hosting two animals and observed daily for the week following the irradiation and then weekly. Body weight and neurological observation (in particular signs of contralateral hemiparesis) were performed once a week (Fig. 4). Motor behavior was assessed by the rotarod test. The rotarod apparatus consisted of a rotating horizontal cylinder (30 mm) and a motor driver control unit (Ugo Basile, Varese, Italy). The cylinder was divided into five separate rotating compartments and fully enclosed to ensure that the rats did not jump out of their area. Rats were placed on the rod, which was rotating at an accelerating speed from 5 to 15 rpm. Automatic timers recorded the time (in seconds) the rats remained on the rod. Control (non-irradiated) and irradiated rats were assessed 7 days, 1, 2, 3 and 4 months after irradiation. In each day of testing rats were placed on the rotarod apparatus three times.

### Evaluation of post-irradiation behavior in rats undergoing motor cortex microbeam transections after local kainic acid injection

Rats were observed for signs of seizures. Seizures were classified as non-convulsive limbic seizures (staring, automatisms) and convulsive seizures (unilateral forelimb clonus, bilateral forelimb clonus, generalized tonic-clonic seizures). After KA injection continuous observation was performed by 3 skilled observers in alternating blocks of 8 hours during the first 24 hours. During the second and third day time, continuous observation was done in blocks of 6 hours. Then the rats underwent observation for 1 hour once a day for 4 days and, after the first week, weekly observations of 1 hour. The period between the first and last observed convulsive seizure was annotated.

### Immunohistological evaluation of the short and long term effects of cortical transections in naïve rats

Two days after irradiation, 2 out of 12 naïve rats receiving motor cortex transections were randomly chosen and sacrificed. Brains were rapidly dissected out and 20 µm coronal sections were cut at −20°C on a cryostat (Microm HM560, Walldorf, Germany). For immunohistochemistry, sections were fixed with PFA 4% for 15 min and blocked with donkey normal serum (DNS) diluted in phosphate-buffered saline (PBS) for 1 hour (PBS/DNS 5%). As marker of DNA damage, brain sections were pretreated with alcohol 50% in PBS for 1 hour at room temperature and then incubated with the primary antibody against phosphorylated H2AX (1∶500, Upstate Biotecnology, Lake Placid, NY, USA). Sections were washed 4 times with PBS, then incubated with the secondary antibodies Alexa Fluor-conjugated donkey F(ab′)2 (1∶200, Invitrogen, Carlsbad, CA, USA) for 2 hours at room temperature. The sections were examined with a Nikon Eclipse E600 microscope equipped for epifluorescence. The remaining rats were killed by decapitation and brains were dissected out and immediately frozen at −80°C. Ten micron thick serial sections were cut and used for histological analysis after having been stained with cresyl violet for Nissl.
